# Gene Reactivation by 5-Aza-2′-Deoxycytidine–Induced Demethylation Requires SRCAP–Mediated H2A.Z Insertion to Establish Nucleosome Depleted Regions

**DOI:** 10.1371/journal.pgen.1002604

**Published:** 2012-03-29

**Authors:** Xiaojing Yang, Houtan Noushmehr, Han Han, Claudia Andreu-Vieyra, Gangning Liang, Peter A. Jones

**Affiliations:** 1 Department of Urology, Norris Comprehensive Cancer Center, Keck School of Medicine, University of Southern California, Los Angeles, California, United States of America; 2 USC Epigenome Center, University of Southern California, Los Angeles, California, United States of America; 3 Department of Pharmacology and Pharmaceutical Sciences, School of Pharmacy, University of Southern California, Los Angeles, California, United States of America; The Babraham Institute, United Kingdom

## Abstract

5-Aza-2′-deoxycytidine, approved by the FDA for the treatment of myelodysplastic syndrome (MDS), is incorporated into the DNA of dividing cells where it specifically inhibits DNA methylation by forming covalent complexes with the DNA methyltransferases (DNMTs). In an effort to study the correlations between DNA methylation, nucleosome remodeling, and gene reactivation, we investigate the integrated epigenetic events that worked coordinately to reprogram the methylated and closed promoters back to permissive chromatin configurations after 5-Aza-2′-deoxycytidine treatment. The ChIP results indicate that H2A.Z is deposited at promoter regions by the Snf2-related CBP activator protein (SRCAP) complex following DNA demethylation. According to our genome-wide expression and DNA methylation profiles, we find that the complete re-activation of silenced genes requires the insertion of the histone variant H2A.Z, which facilitates the acquisition of regions fully depleted of nucleosome as demonstrated by NOMe–seq (Nucleosome Occupancy Methylome–sequencing) assay. In contrast, SRCAP–mediated H2A.Z deposition is not required for maintaining the active status of constitutively expressed genes. By combining Hpa II digestion with NOMe–seq assay, we show that hemimethylated DNA, which is generated following drug incorporation, remains occupied by nucleosomes. Our data highlight H2A.Z as a novel and essential factor involved in 5-Aza-2′-deoxycytidine–induced gene reactivation. Furthermore, we elucidate that chromatin remodeling translates the demethylation ability of DNMT inhibitors to their downstream efficacies, suggesting future therapeutic implications for chromatin remodelers.

## Author Summary

Epigenetic changes, which include chemical modifications to the DNA and changes in the proteins that package DNA to fit into a cell, play an important role in gene expression regulation. The fact that a number of abnormal epigenetic changes that lead to the silencing of genes occur during tumorigenesis has prompted the design of epigenetic therapies. The ultimate goal of these therapies is to reverse the aberrant epigenetic modifications observed in cancer cells, thereby restoring cells to a “normal” state. 5-Aza-CdDR, a FDA approved drug for MDS treatment, reverses a chemical modification of the DNA resulting in gene reactivation. The data presented here show the importance of H2A.Z, a special DNA packaging protein variant, in the gene reactivation process induced by 5-Aza-CdR. The presence of H2A.Z facilitates the access of proteins at gene regulatory regions, which is a necessary step for gene re-expression. A better understanding of the events that follow 5-Aza-CdR treatment is a necessary step towards the design of combination and/or personalized epigenetic therapies.

## Introduction

The eukaryotic genome is compacted into chromatin and associated proteins. The fundamental repeating unit of chromatin is the nucleosome, which contains ∼147 bp of DNA wrapped around a histone protein octamer [1]. However, chromatin conformations change during various cellular processes, such as the cell cycle, transcription or DNA damage [2]. During gene activation, transcription factors compete with chromatin packaging proteins in order to gain access to the DNA sequence and read the genetic information accurately. Accumulated evidence shows that the chromatin architecture of gene promoter regions strongly regulates gene transcription [3]. This chromatin environment might be altered by DNA methylation, post-translational modifications of histone proteins, histone variants and nucleosome positioning [4].

In mammalian cells, ∼60% of gene promoters are located within CpG islands, where cytosine methylation of CpG dinucleotides impairs gene expression. Histone modifications and histone variants are also strongly correlated with transcriptional status [3]. Nucleosome positioning plays an essential role in gene transcriptional regulation according to recent genome wide studies, which show that the majority of active or poised promoters have decreased nucleosome density [5]. Furthermore, the histone variants H2A.Z and H3.3, which are located at specific genome regions such as promoters, enhancers and insulators, work coordinately to destabilize nucleosomes [6]–[8]. The ATP dependent nucleosome remodelers catalyzing H2A.Z incorporation, namely SRCAP and p400 complexes in mammalian cells, have been suggested to be involved in transcriptional regulation, however, the role of H2A.Z remains controversial [9]–[15].

Abnormalities in epigenetic modifications play an essential role in tumorigenesis [16], and the reversal of them is the basic concept of epigenetic therapy for cancer. DNA methyltransferases (DNMT inhibitors), such as 5-azacytidine (5-Aza-CR) and 5-Aza-2′-deoxycytidine (5-Aza-CdR), are approved by the FDA for the treatment of MDS [17]–[18]. Although CpG demethylation is the direct and immediate consequence of treatment with DNMT inhibitors (5-Aza-CR and 5-Aza-CdR) [19], the level of demethylation in tumor suppressor genes does not predict clinical outcome, which suggests that unknown biological processes link the demethylation effects of DNMT inhibitors to their clinical benefits [20]. Several reports have already shown that, in addition to CpG demethylation, DNMT inhibitors indirectly reduce some repressive histone marks, increase acetylation of histone H3 and promote nucleosome depletion upstream of the transcription start sites (TSS) [21]–[24].

Here, taking advantage of a high resolution nucleosome positioning assay developed by our laboratory, we further study the integrated epigenetic changes following 5-Aza-CdR induced demethylation. In addition to the rapid enrichment of H3K4me3 at promoter regions, we find that H2A.Z incorporation increases in response to demethylation. Notably, CpG demethylation induced enrichment of H2A.Z and H3K4me3, as well as nucleosome depletion coordinately constitute a “permissive” chromatin architecture independently of histone acetylation levels. Inhibiting H2A.Z deposition by SRCAP knockdown lessens the establishment of “permissive” promoter environments and ultimately reduces the levels of gene reactivation after 5-Aza-CdR treatment. Genome-wide gene expression and DNA methylation studies further confirm that SRCAP-mediated H2A.Z insertion promotes DNA demethylation induced gene re-expression but has minimal effects on constitutively active genes. Our study reveals an important function of SRCAP/H2A.Z in promoting the reactivation process induced by 5-Aza-CdR but not in maintaining the expression of constitutively active genes and provides an insight to the chromatin structure of hemimethylated DNA.

## Results

### 5-Aza-CdR induces activation of methylation silenced genes by demethylating DNA and changing chromatin structure around promoter regions

**Figure 1:**
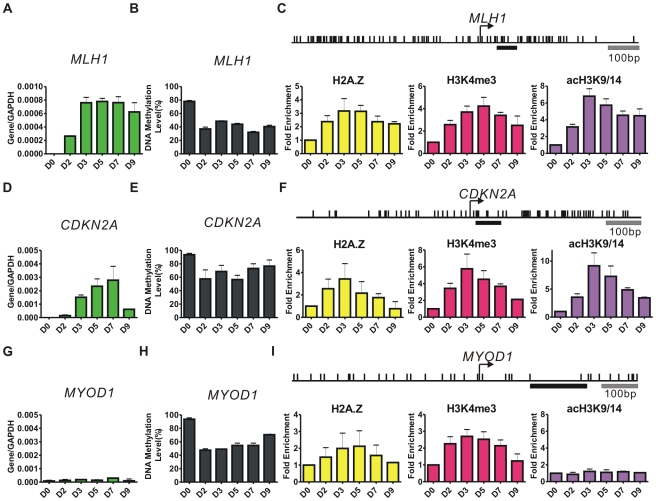
5-Aza-CdR induces dynamic changes in gene expression, DNA methylation, and histone marks. A, D, G, RT-PCR results showing the expression levels of *MLH1*, *CDKN2A* and *MYOD1* at the indicated days (D) after 1 uM 5-Aza-CdR treatment. The mRNA levels were normalized to *GAPDH*. Error bars represent the standard deviation of biological triplicates. B, E, H, DNA methylation levels at promoters were measured by Ms-SNuPE. Error bars represent the range between biological duplicates. C, F, I, ChIP results of histone variants and modifications were normalized to histone H3 at the indicated time points after treatment. Arrows indicate TSSs and the upper vertical bars represent CpG sites. Underlying bold horizontal lines indicate the ChIP regions amplified by PCR. Results from three independent biological experiments of H2A.Z and two independent biological experiments of H3K4me3 (acH3K9/14) are shown.

To investigate the effects of DNA demethylation on chromatin architecture and gene expression, we treated the RKO colon cancer cell line, with 1 uM 5-Aza-CdR for 24 hours and followed the sequential changes in mRNA expression, DNA methylation and histone marks at the promoters of the *MLH1*, *CDKN2A* and *MYOD1*, which are methylated and silenced in RKO cells (Figure 1).

*MLH1* expression began to rise at D2 after 5-Aza-CdR treatment, reaching a maximal level at D3 and remained constant for 4 days (Figure 1A). We performed a quantitative Methylation-sensitive Single-Nuleotide Primer Extension (Ms-SNuPE) assay to detect the DNA methylation changes at the indicated days (Figure 1B). A striking decrease in DNA methylation was observed at D2 (∼40%). The methylation level at the *MLH1* promoter remained nearly constant from D2 to D9. We then used ChIP to monitor the changes of histone marks after 5-Aza-CdR treatment (Figure 1C). The enrichments of H2A.Z, H3K4me3 and H3K9/14 acetylation (acH3K9/14) were normalized to histone H3 levels to eliminate the potential influence of nucleosome depletion after drug treatment, and the ChIP primers were designed to amplify stable nucleosome regions which located just downstream of TSSs [5]. Interestingly, our results showed that the H2A.Z enrichment significantly changed after 5-Aza-CdR treatment (p<0.001), and could be observed as early as D2 when DNA methylation was substantially decreased (Figure 1C). H3K4me3 increased immediately after treatment and the enrichment of acH3K9/14 modestly increased at D2 and peaked by D3 displaying a similar pattern to the levels of *MLH1* expression (Figure 1A).

The mRNA levels of *CDKN2A* rose steadily from D2 after 5-Aza-CdR treatment and peaked at D7 but abruptly dropped at D9 (Figure 1D). Although the active histone marks increased at the promoter of *CDKN2A* in a manner similar to *MLH1*, the H2A.Z level (p<0.05) diminished nearly to the baseline at D9 along with a rapid decline of acH3K9/14 from D5 to D9 (Figure 1F).

The mRNA level of *MYOD1*, a self-regulated gene expressed exclusively in muscle cells, remained undetectable and showed no RNA polymerase II (pol II) enrichment after 5-Aza-CdR treatment even though it showed a demethylation pattern similar to that of *MLH1* (Figure 1G, 1H; Figure S1A). Most interestingly, we observed modest changes in H2A.Z and H3K4me3 at the *MYOD1* promoter, whereas acH3K9/14 remained extremely low, in agreement with its low expression level (Figure 1I). Therefore the *MYOD1* promoter acquired a “permissive” state for expression after 5-Aza-CdR treatment but the gene was not expressed.

In addition to analyzing DNA methylation changes at the single strand level (Figure 1B, 1E, 1H), we detected and quantified asymmetrically methylated DNA after 5-Aza-CdR treatment. We performed a hemimethylation Ms-SNuPE assay based on Hpa II digestion as we have developed previously and used the *CDKN2A* gene as a model (Figure S1B-S1D) [25]. The majority of DNA molecules were hemimethylated DNA duplexes at D2 (∼60%). Even at D5, ∼5% of the DNA duplexes were composed of hemimethylated DNA molecules. There was a small portion of fully demethylated DNA duplexes at D2, however, the maximal levels of double strand demethylation (∼40%) was detected at D3–5 when hemimethylated DNA levels dropped.

Collectively, our results demonstrate that 5-Aza-CdR treatment eventually produces two types of demethylated DNA duplexes, hemimethylated and fully demethylated DNA. After demethylation H2A.Z and H3K4me3 are deposited in all three promoters. Interestingly, only acH3K9/14 shows a correlation between its enrichment and mRNA expression.

### Nucleosome occupancy is disrupted by 5-Aza-CdR treatment

**Figure 2:**
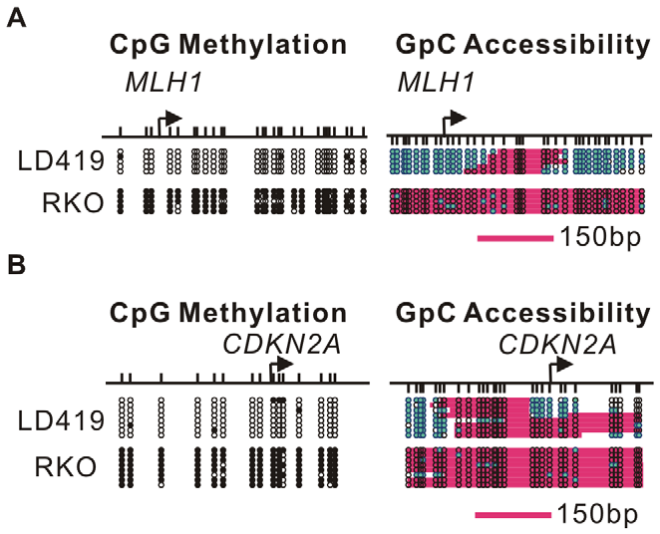
DNA methylation influences nucleosome occupancy. A. Nucleosome occupancy and DNA CpG methylation levels at the *MLH1* promoter in LD419 cells (upper) and RKO cells (lower) were investigated by NOMe-seq. B. Nucleosome occupancy and DNA CpG methylation levels at the *CDKN2A* promoter in LD419 cells (upper) and RKO cells (lower) were examined by NOMe-seq. The upper vertical bars represent CpG sites and the lower vertical bars indicate GpC sites. Open and filled circles represent unmethylated and methylated CpG sites respectively. The teal filled circles indicate GpC sites which are methylated and therefore accessible to GpC methyltransferase. Pink areas represent regions which are resistant to GpC methyltransferase and longer than 146 bp.

**Figure 3:**
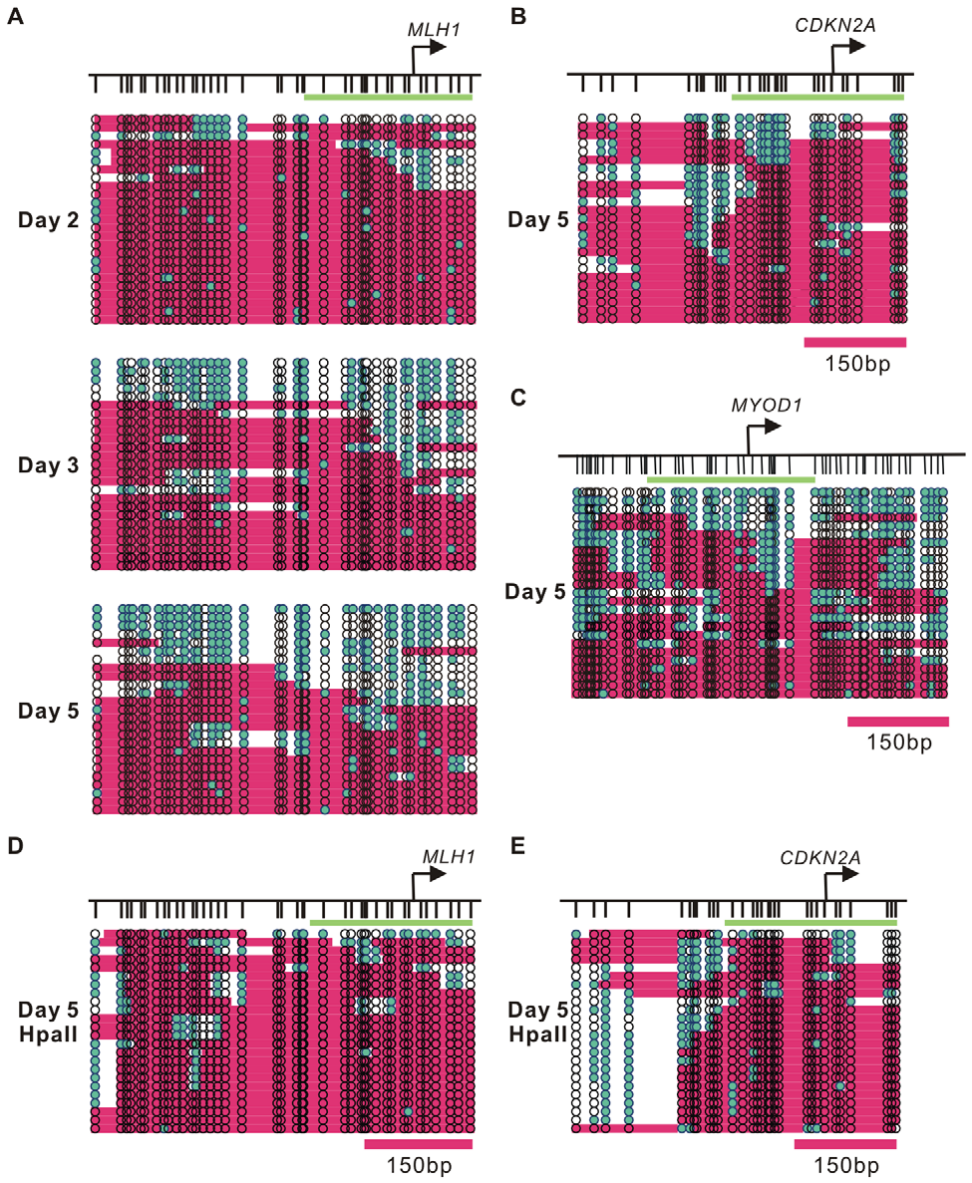
Dynamic nucleosome occupancy changes on symmetrically and asymmetrically methylated DNA duplexes after 5-Aza-CdR treatment. A. The nucleosome occupancy changes at the *MLH1* promoter after 5-Aza-CdR treatment were investigated by NOMe-seq assay. The GpC methyltransferase accessibility on each strand is shown. B. Nucleosome depleted regions were detected at the *CDKN2A* promoter five days after 5-Aza-CdR treatment. C. Nucleosome depleted regions were also detected in the *MYOD1* promoter five days after 5-Aza-CdR treatment. D,E. The nucleosome occupancy on asymmetrically methylated DNA duplexes was analysis by NOMe-seq after Hpa II digestion at the *MLH1* and *CDKN2A* promoters. Green bars presents regions of 250 bp in length, which covers the −1 nucleosome plus 100 bp downstream of that nucleosome.

We next investigated the effects of 5-Aza-CdR on nucleosome occupancy (Figure 2, Figure 3). We previously examined the nucleosome occupancy status of the *MLH1* promoter in RKO and LD419 cells using MNase-ChIP assay and Methylase-based Single-Promoter Analysis assay (MSPA) [22]. We confirmed our previous findings using a recently developed high resolution assay, which uses the GpC methyltransferase (M.CviPI) instead of CpG methyltransferase (M.SssI) to methylate GpC sites that are not occupied by nucleosomes or tightly bound transcription factors [26]. By analyzing the methylation status of GpC sites, this NOMe-seq (Nucleosome Occupancy Methylome-sequencing) assay provides a digital footprint of nucleosome occupancy and allows the study of nucleosome positioning in both CpG islands and CpG poor regions regardless of their CpG methylation status.

Nucleosome occupancy at the *MLH1* and *CDKN2A* promoters in LD419 and RKO cells were analyzed by NOMe-seq using PCR primers lacking CpG or GpC sites to avoid complications due to cytosine methylation in the parental molecules (Figure 2). Both promoters were unmethylated in LD419 cells and had clear nucleosome depleted regions (NDRs) which were accessible to the exogenous M.CviPI. In contrast, the promoters of *MLH1* and *CDKN2A* were methylated in RKO cells and were inaccessible to the GpC methyltransferase, indicating that hypermethylated promoters were fully occupied by nucleosomes.

To investigate the nucleosome occupancy changes accompanying drug induced DNA demethylation, we used primers which were specifically designed to amplify the DNA strands which had become demethylated at CpG sites and studied the accessibility of these demethylated molecules to M.CviPI (Figure 3; Figure S2). The maximum demethylation of CpG sites was observed at D2 (Figure 1B), but only one out of twenty-five demethylated DNA strands (4%) had a nucleosome depleted region larger than 146 bp around the TSS (green bar on the graph)(Figure 3A). At D3, a proportion of unmethylated DNA strands at the gene promoters (24%) showed a nucleosome depleted area large enough to accommodate at least one nucleosome, consistent with the presence of active histone marks and increased gene expression. Extensive nucleosome depletion (40%), the H2A.Z enrichment and gene reactivation reached a maximal level at D5 (Figure 3A). NOMe-seq analysis of the *CDKN2A* promoter yielded similar results and showed depletion of the −1 nucleosome at D5 (12%) (Figure 3B). Interestingly, the *MYOD1* promoter showed drug-induced enrichment of H2A.Z and H3K4me3 as well as nucleosome depletion around the TSS (20%) without *MYOD1* expression (Figure 3C). Therefore, changes in histone modifications and nucleosome depletion were the direct consequences of DNA demethylation and did not require transcriptional activation for some genes, such as *MYOD1*.

Although the nucleosome occupancy at the *MLH1* promoter was dramatically decreased on the demethylated DNA strands at D5 after 5-Aza-CdR treatment, a portion of demethylated DNA strands remained inaccessible to M.CviPI (Figure 3A). As shown in Figure S1C, the majority of demethylated DNA strands were associated with hemimethylated DNA duplexes at D2. And the demethylated DNA strands at the D2 were highly occupied by nucleosomes. To further investigate the nucleosome occupancy on hemimethylated DNA, we pre-digested the NOMe-seq DNA samples before bisulfite treatment with an excess of Hpa II. Demethylated DNA strands associated with symmetrically demethylated DNA duplexes are destroyed by Hpa II digestion, whereas the demethylated strands in hemimethylated DNA duplexes are resistant to digestion (Figure S1B). Next, we used the same PCR primers as shown previously to amplify the remaining demethylated DNA strands. The NOMe-seq results from Hpa II digested DNA clearly showed that the promoters of *MLH1* and *CDKN2A* were occupied by nucleosomes when the underlying DNA was hemimethylated (Figure 3D, 3E). Our results show that DNA demethylation at promoter regions induces substantial changes in nucleosome occupancy which only occurs on symmetrically demethylated but not hemimethylated DNA.

### SRCAP–mediated H2A.Z incorporation promotes demethylation induced gene expression and nucleosome depletion

**Figure 4:**
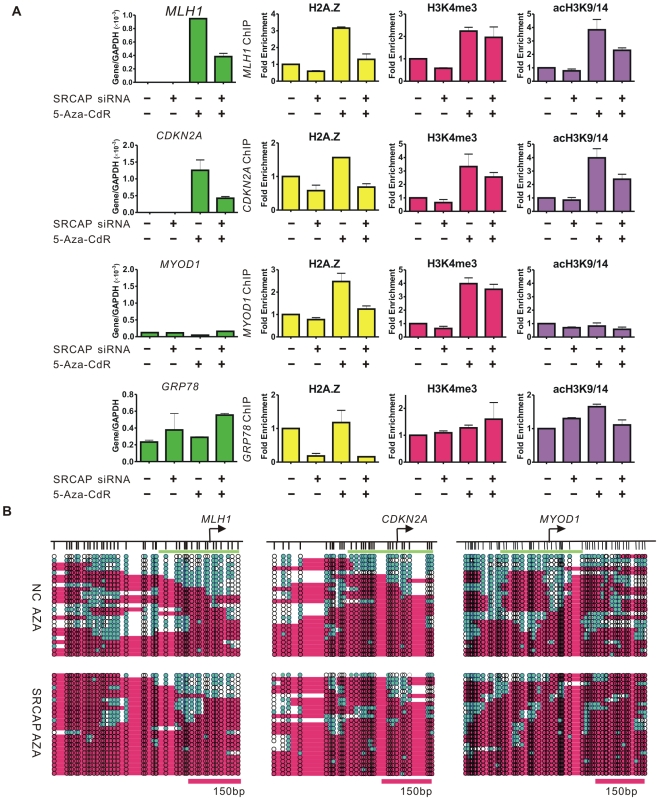
SRCAP knockdown prevents 5-Aza-CdR–induced H2A.Z deposition and nucleosome remodeling. A, RKO cells were treated with the indicated siRNA for 24 hours followed by treatment of 1 uM 5-Aza-CdR (+) or PBS (−) for another 24 hours. RNA was isolated 72 hours after drug treatment. The gene expression levels were measured by RT-PCR and the data represent the means of biological triplicates. The enrichment of histone marks after the indicated treatment was measured by ChIP and normalized to the Histone H3 level. The data represent biological duplicates. B. NOMe-seq results show the nucleosome occupancy at the *MLH1*, *CDKN2A* and *MYOD1* promoter after the indicated treatment.

The enrichment of H3K4me3 after demethylation has been well studied [27], however the role of H2A.Z insertion in gene reactivation is unclear. Thus, we explored the potential role of H2A.Z in 5-Aza-CdR induced gene reactivation by knocking down SRCAP (Figure S3A), which catalyzed H2A.Z deposition in a cell cycle independent manner [28], and subsequently treating the cells with 5-Aza-CdR (Figure 4A).

The expression of *MLH1* and *CDKN2A* was strongly attenuated by SRCAP knockdown, concomitant with a reduction of H2A.Z levels at the promoters (Figure 4A). The enrichment of acH3K9/14 at the reactivated promoters was reduced after SRCAP knockdown as well. Interestingly, the 5-Aza-CdR induced H2A.Z deposition was also inhibited by SRCAP knockdown at the *MYOD1* promoter, but the gene expression and acH3K9/14 levels remained undetectable as expected. Of note, SRCAP knockdown showed minimal effects on the H3K4me3 levels at all three promoters, suggesting that the H3K4me3 mark was independent of H2A.Z levels. In addition, knockdown of SRCAP did not affect DNA methylation levels at promoters of these examined genes (Figure S3B).

In contrast, the mRNA level of *GRP78*, which is usually over-expressed in cancer cells and has enriched H2A.Z around its promoter [2], was not reduced and even modestly increased after SRCAP knockdown. The H2A.Z level at the *GRP78* promoter dramatically dropped by nearly 90% compared with non-target (NC) siRNA treated cells. Meanwhile, the levels of H3K4me3 and acH3K9/14 remained high showing that these marks were independent of H2A.Z levels. In addition to *GRP78*, we analyzed two more genes, *LAMB3* and *G3BP*, both of which were unmethylated and expressed in RKO cells (Figure S3D, S3E). The enrichment of H2A.Z, which has been identified at both promoters [10], [29], was reduced by SRCAP knockdown, but neither the mRNA expression nor the histone marks were significantly affected. Remarkably, knockdown of SRCAP in LD419 cells did not inhibit the expression of *MLH1* and *CDKN2A*, though the H2A.Z enrichment at the promoter regions had been reduced. Again, no difference in H3K4me3 and acH3K9/14 levels was detected after SRCAP siRNA treatment (Figure S4A).

We next investigated the function of SRCAP-mediated H2A.Z deposition on 5-Aza-CdR induced nucleosome occupancy changes. Substantial nucleosome depletion at the *MLH1* promoter was observed on the demethylated DNA strands in the NC siRNA treated cells as previously shown (Figure 4B). When SRCAP-mediated H2A.Z incorporation was inhibited, nucleosome depletion was much curtailed at the promoter (32% to 20%). Similarly, we observed that inhibiting SRCAP-mediated H2A.Z deposition prevented the depletion of nucleosome induced by the 5-Aza-CdR in the vicinity of the *CDKN2A* (20% to 4% ) and *MYOD1* (16% to 8%) TSSs. In contrast, the NDRs at the *GRP78*, *LAMB3* and *G3BP* promoters were not reduced and even showed modest increases (Figure S3C, S3F, and S3G). Similarly, the NDRs upstream of the TSS of *MLH1* did not change after SRCAP knockdown in LD419 cells (Figure S4B).

Taken together, these results demonstrate that SRCAP-mediated H2A.Z deposition and associated nucleosome depletion play a key role in re-constructing a poised chromatin architecture around demethylated promoters. In contrast, continued H2A.Z presence is not critical in maintaining an open chromatin environment of actively transcribed genes.

### Knockdown of SRCAP alters the expression profile of 5-Aza-CdR–activated genes but has minimal effects on DNA methylation patterns

To elucidate the importance of H2A.Z deposition for gene reactivation following 5-Aza-CdR induced demethylation, we conducted genome-wide studies to assay global DNA methylation and gene expression changes after drug treatment.

**Figure 5:**
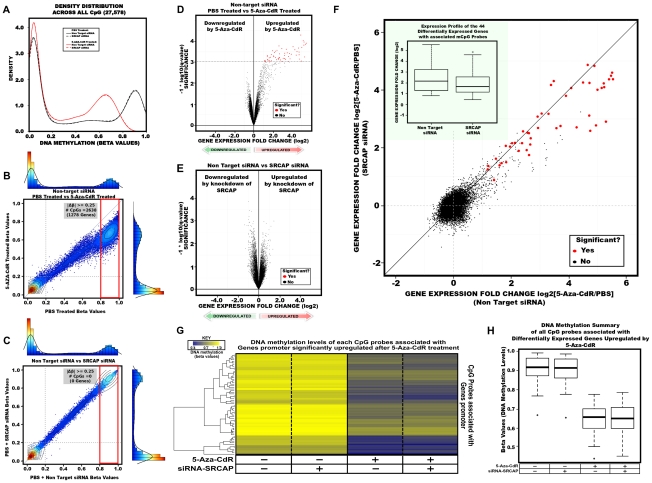
DNA methylation and gene expression changes globally after 5-Aza-CdR treatment and SRCAP knockdown. A. Density plots for each sample across all 27,578 CpG sites analyzed. The X-axis represents beta values ranging from 0 (no DNA methylation) to 1 (high DNA methylation). Black colored lines represent samples treated with PBS and red lines are samples treated with 5-Aza-CdR. Solid lines are NC siRNA treated samples and dashed lines are SRCAP siRNA treated samples. B,C. Scatter plot overlayed with histogram and density distribution. Each dot represents an interrogated CpG probes beta value. Colored dots represents density or number of probes as indicated in the adjacent axis as illustrated as a both a histogram and density distribution. Contour lines are drawn to further illustrate the number of probes for a specified region. B. Represents a scatter plot between 5-Aza-CdR vs PBS treatment (control) in NC siRNA treated RKO cells. C. Represents a scatter plot between SRCAP siRNA vs NC siRNA treated cells. D,E . The gene expression log2 fold difference is plotted on the x-axis, and the q-value which accesses significance is plotted on the y-axis (−1* log10 scale). Probes that are identified as significantly different between two groups are colored in red. D. NC 5-Aza-CdR vs NC PBS; E. NC vs SRCAP siRNA F. Scatter plot of all transcript probes assayed on the Illumina Human expression beadchip. Probes identified as differentially expressed and demethylated after 5-Aza-CdR treatment in non-target siRNA treated cells (x-axis) are colored red. Inset shows the distribution (box plots) of the demethylation induced gene reactivation fold changes in non-target siRNA and SRCAP siRNA treated cells. G. One-dimensional hierarchical clustering of the demethylated CpG probes which interrogate the promoters of genes significantly upregulated by 5-Aza-CdR treatment. Each row represents a probe; each column represents a sample. The level of DNA methylation (beta value) for each probe in each sample is represented by using the color scale shown in the legend; white indicates missing data. H. Box plot of DNA methylation levels distribution for genes which were significantly upregulated and demethylated by 5-Aza-CdR.

We interrogated global promoter DNA methylation patterns using the Infinium HumanMethylation27 platform, which includes 27,578 CpG dinucleotides spanning 14,495 well-annotated, unique gene promoter and/or 5′ gene regions (from −1,500 to +1,500 from the TSS). The DNA methylation level for each interrogated CpG site is reported as a beta value, ranging from zero (low DNA methylation) to one (high DNA methylation). In NC siRNA treated cells, the CpG sites could be roughly separated into two groups based on the bimodal distribution of the beta values: a hypomethylated group (beta value<0.2) and a hypermethylated group (beta value>0.8) [30] (Figure 5A). Consistent with the Ms-SNuPE results (Figure 1B, 1E, 1H), the CpG sites within the promoters of *MLH1*, *CDKN2A* and *MYOD1* had beta values higher than 0.8. The peak representing hypermethylated probes was notably shifted towards the left after 5-Aza-CdR treatment. To further expand this observation, CpG probes were plotted between 5-Aza-CdR and PBS treatment (control) in NC siRNA treated cells (Figure 5B). Using a beta value difference of 0.25 as a threshold for differential DNA methylation and separating CpG probes based on beta values>0.8 associated with control treatment, 2,638 CpG probes (1278 genes) were identified to be demethylated by 5-Aza-CdR in NC siRNA treated cells and knockdown of SRCAP only showed little effect on DNA methylation patterns, as 2,515 CpG probes (1208 genes) were found to be demethylated in SRCAP siRNA treated cells (Figure 5C; Figure S5A, S5B).

To evaluate gene expression changes, we performed a permutation analysis (1,000 permutations) using Significance Analysis of Microarrays (SAM) in NC or SRCAP siRNA transfected cells after 5-Aza-CdR treatment [31]. The identification of differentially expressed genes was performed among the indicated groups (Figure 5D–5F; Figure S5C, S5D). We found that SRCAP knockdown had minimal impact on global gene expression (Figure 5E), while 5-Aza-CdR treatment significantly up-regulated 97 genes (representing 130 different transcripts) in NC treated cells and 86 genes (representing 105 different transcripts) in SRCAP siRNA treated cells, with an 81% overlap between two groups (Figure 5D; Figure S5E, S5F). We did not observe any gene significantly down-regulated by 5-Aza-CdR treatment in either NC or SRCAP siRNA treated cells.

To visualize the global gene expression difference between SRCAP and NC siRNA, we plotted the observed log2 fold change for all the interrogated transcripts on the platform (Figure 5F). After extracting the promoter DNA methylation beta values of 97 genes, which were reactivated by 5-Aza-CdR in NC siRNA treated cells from the Infinium array, we found that 44 genes (representing 92 different CpG loci), including *MLH1* and *CDKN2A*, had beta value differences greater than 0.2 as shown in the heatmap and box plot (Figure 5G, 5H). We next concentrated on these 44 genes which were demethylated and subsequently reactivated. Within this group of genes, knockdown of SRCAP significantly inhibited the reactivation of some transcripts such as *EPM2AIP1* from up-regulated to non-responsive. Although the majority of genes that were up-regulated by 5-Aza-CdR in NC siRNA treated cells were still induced in SRCAP siRNA treated cells, the reactivation levels were strikingly decreased (red circles). The fold changes of the 44 genes induced by demethylation were calculated in the inserted box plot, further showing the significant effects of SRCAP knockdown (p<2×10^−16^). To validate this genome-wide analysis, we randomly selected four candidate genes (*CHFR*, *CTCFL*, *SYCP3* and *EPM2AIP1*) from the pool of 44 genes and analyzed the expression changes (Figure S6A–S6C). The reactivation levels of four methylated genes were significantly suppressed by SRCAP knockdown. We then validated the histone marks changes on the promoters of *CHFR* and *SYCP3*. We found that SRCAP knockdown prevented H2A.Z deposition as well as diminished gene reactivation, which was consistent with our results from *MLH1* and *CDKN2A*. To confirm that the H2A.Z insertion was causing the observed effects, we knocked down p400, which is a homolog of SWR1 and has been identified as another key player in H2A.Z deposition [13]. We found that inhibiting p400 could also reduce the reactivation of *MLH1* and *CDKN2A* in RKO cells (Figure S6D). In addition to knocking down the two catalytic subunits of SRCAP and p400 complexes, inhibition of YL-1, the binding partner of H2A.Z in the SRCAP complex [32]–[33], also suppressed 5-Aza-CdR induced gene reactivation (6E) and depletion of the −1 nucleosome (Figure S6F).

Our integrated study reveals that 5-Aza-CdR robustly reduces global promoter DNA methylation levels, and subsequently reactivates gene expression. Decreasing SRCAP expression inhibits global gene reactivation but has no effect on DNA methylation at promoter. However the maintenance of active gene expression might not require highly enriched H2A.Z.

## Discussion

**Figure 6:**
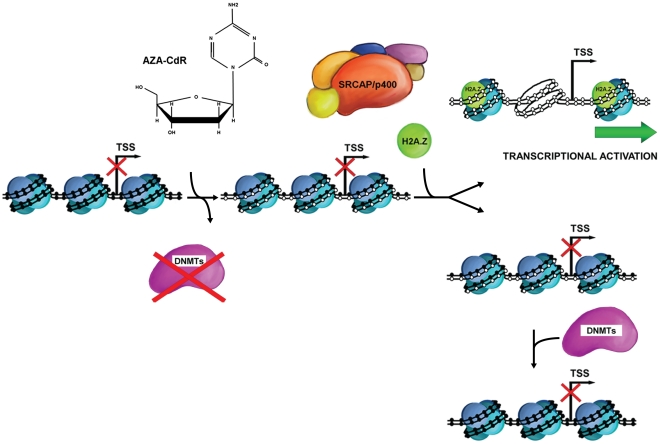
Simplified schematic of 5-Aza-CdR–induced DNA demethylation and chromatin configuration changes. During DNA replication, 5-Aza-CdR is incorporated into DNA, sequesters DNMTs immediately and this results in demethylation. After a cycle of cell doubling, the majority of DNA molecules are hemimethylated and occupied by nucleosomes. Subsequent DNA replications produce more symmetrically demethylated DNA molecules, possessing NDRs around the TSS. In addition, DNA demethylation results in SRCAP-mediated H2A.Z deposition at gene promoter, which promote gene reactivation.

Although recent studies have begun to explore the epigenetic factors involved in the 5-Aza-CdR mediated demethylation process [21], [24], our study focuses on the dynamic changes in chromatin architecture immediately after 5-Aza-CdR treatment (Figure 6). We demonstrate that removing DNA methylation rapidly induces H2A.Z incorporation, which confirms the antagonistic relationship between H2A.Z and DNA methylation observed in genome-scale studies of *arabidopsis*, human breast tissue and tumorigenesis of a B-cell lymphoma model in mouse [34]–[36]. Although some reports show that loss of *pie1* in *Arabidopsis* or H2A.Z in mammals increases DNA methylation levels at gene body regions [34], [37], our data demonstrate that DNA methylation levels at promoter regions are not affected by transiently inhibiting H2A.Z insertion. Previous reports have showed positive correlations between H2A.Z insertion and the expression of *CDKN1A*, estrogen receptor target genes, muscle differentiation-specific genes and PcG protein target genes in ES cells [11], [13], [33]. Our genome-wide expression results demonstrate that SRCAP-mediated H2A.Z deposition at promoter regions is necessary for complete gene reactivation induced by DNA demethylation. We show that inhibition of SRCAP-mediated H2A.Z insertion prevents nucleosome depletion at the promoters of *MLH1* and *CDKN2A* after 5-Aza-CdR treatment. In addition, knockdown of YL-1, the binding partner of H2A.Z in the SRCAP complex, also reduces the *CDKN2A* gene reactivation and nucleosome depletion around the TSS region that are induced by 5-Aza-CdR treatment. Collectively, our data provides evidence for the hypothesis that SRCAP/H2A.Z directly promotes transcription by reducing nucleosome occupancy at promoter regions [38]. Nevertheless, H2A.Z enrichment is necessary but not sufficient for gene reactivation according to our data. As shown at the *MYOD1* promoter, the modest enrichment of H2A.Z contributes to the establishment of a “permissive” environment regardless of the subsequent gene reactivation status. The reduction of M.CviPI accessibility at the *MYOD1* promoter after SRCAP knockdown suggests that H2A.Z mediated nucleosome depletion is not the consequence of gene expression and might be an early event in transcription initiation. In addition, Hardy *et al* [15] showed that H2A.Z was recruited to the promoter regions prior to pol II binding. We observed pol II enrichment at the *MLH1* promoter but not at the *MYOD1* promoter, though both promoters have been remodeled structurally, which suggested the formation of “permissive” promoter regions might not require the presence of pol II at an early stage.

Unlike H2A.Z, histone H3 acetylation occurs concomitantly with gene expression, and is not required for establishing this early “permissive” promoter. Furthermore, the data from the SRCAP knockdown experiments strongly indicate that H2A.Z incorporation, especially SRCAP mediated deposition, is independent of H3K9/K14 acetylation. Functional studies of the Tip48/49 complex , which shares some components of the SRCAP complex, showed that acetylation of H2A enhanced H2A.Z insertion [39]. However, a recently published report demonstrated that inhibiting NF-Y, one of the proteins with highly similarity to core histones, prevented H2A.Z deposition at promoter regions but had no observable effect on histone H3K9/14 acetylation, indirectly supporting our conclusions [40].

Similarly to the behavior of H2A.Z, H3K4me3 enriches at promoters following demethylation, in agreement with the reported mutually exclusive relationship between H3K4me3 and DNA methylation [41]. Although many reports show that H3K4me3 levels are correlated with gene expression status, Thomson et al [42] demonstrated that artificially inserted promoter-less DNA sequences containing unmethylated CpG sites were sufficient to acquire H3K4me3. In our study, DNA demethylation associated H3K4me3 enrichment creates a “permissive” promoter configuration; however, such a configuration is not sufficient for gene activation. The presence of key transcription factors is also necessary [29]. In yeast, Set1, an H3K4 methyltransferase, and H2A.Z have redundant functions in preventing the spread of Sir-mediated silencing, indicating that the presence of H3K4 methylation marks and H2A.Z are not dependent on each other [43]. Interestingly, our results show that the enrichment of H3K4me3 is not affected after inhibiting SRCAP-mediated H2A.Z deposition and suggest that in mammalian cells the regulation of these histone marks might not depend on each other. Therefore, it would be interesting to study the specific effects of H3K4me3 on chromatin remodeling after DNA demethylation in the future.

It has been reported that certain DNA sequences and the binding of pol II or transcription factors influence nucleosome occupancy by different mechanisms [44]–[45]. Recent reports have shown that methylated DNA facilitates nucleosome assembly in vitro, and reciprocally, stable nucleosomes contribute to the establishment and maintenance of DNA methylation [46]–[48]. Here, we apply the now well-established NOMe-seq assay to investigate the correlation between nucleosome positioning and DNA methylation after drug treatment [45], [49]. Our data demonstrate that promoter regions are highly occupied by nucleosomes when DNA duplexes are either symmetrically methylated or hemimethylated in living cells, suggesting the dominant role of DNA methylation in maintaining stable nucleosomes. Using reconstituted histone octamers and single-stranded M13 constructs, Deobagkar *et al* show that hemimethylated DNA prevents chromatin expression [50]. Thus complete nucleosome depletion takes place only on symmetrically demethylated DNA after 5-Aza-CdR treatment, which is probably required for full gene activation. Furthermore, our data have confirmed the feasibility of utilizing NOMe-seq in future to investigate the drug induced nucleosome remodeling globally.

According to our genome-wide analysis, 5-Aza-CdR induces global demethylation yet only a limited number of genes are significantly reactivated, indicating that CpG demethylation and the subsequent establishment of open chromatin architectures are essential but not sufficient to induce gene reactivation. The “permissive” promoters created by 5-Aza-CdR treatment, such as the *MYOD1* promoter, have active histone marks and NDRs, which are needed for assembling of the transcriptional machinery, but other regulatory factors are required to fully reactivate these genes. Therefore, epigenetic modulators which regulate histone marks and nucleosome positioning have strong abilities to promote or impede the pharmacological functions of 5-Aza-CdR. Our results provide a rationale to design clinical trials combining DNMT inhibitors with other anticancer drugs, especially histone deacetylase inhibitors, which facilitate histone acetylation. A comprehensive understanding of the coordinated interplay between epigenetic regulators and 5-Aza-CdR will help explain the drug's clinical outcomes as well as promote the discovery of novel therapeutic targets.

## Materials and Methods

### Cell culture

RKO, a colon cancer cell line, was purchased from ATCC and was maintained in MEM medium with 10% FBS. LD419, a normal human bladder fibroblasts was generated by Dr Louis Dubeau and was maintained in McCoy's 5A supplemented with 20% FBS.

### 5-Aza-CdR treatment and transfection

RKO cells were plated at 2×10^6^ cells/100-mm dish and treated with 1 µM of 5-Aza-CdR (Sigma Chemical Co., St. Louis, MO) for 24 hours. The NC pool siRNA (D-001810-10-05) and the ON-TARGET *plus* siRNA targeting SRCAP (L-004830-00-0005), p400 (L-021272-01-0005) and YL-1 (VPS72 L-020097-00-0005) (Thermo Fisher Scientific Inc.) were transfected into RKO cells 24 hours before 5-Aza-CdR treatment using DharmaFECT™ siRNA transfection reagents (Thermo Fisher Scientific Inc.).

### Real-time RT–PCR

Total RNA was extracted using RNeasy kit (Qiagen) and was converted to cDNA by M-MLV Reverse Transcriptase (Promega) using random primer (Promega). The sequences of gene specific primers and taqman-probs are available upon request. With each set of PCR primers, titrations of known amounts of DNA were included as a standard for quantization.

### Methylation-sensitive single-nucleotide primer extension (Ms-SNuPE)

DNA methylation level was determined by Ms-SNuPE as described previously [51]. Briefly, CpG sites were interrogated for each promoter. The methylation level of each gene is the average of the three CpG sites examined by Ms-SNuPE.

### Chromatin immunoprecipitation (ChIP)

ChIP was performed as described previously [52]. Ten µg of the following antibodies were used: anti-Histone H3 (Abcam), anti-acetylated Histone H3K9/14 (Milipore), anti-H2A.Z (Abcam) and anti-RNA polymerase II(Abcam). Five µl of anti-H3K4me3 (Active Motif) antibody was used. Ten µg of Rabbit IgG (Millipore) was used as a non-specific antibody control. PCR primers are available upon request.

### Nucleosome occupancy methylome-sequencing (NOMe-seq)

Nuclei preparation and GpC Methyltransferase treatment were performed as described previously [26]. Briefly, freshly extracted nuclei were treated with 200 U of GpC methyltransferase for 15 min at 37°C. An equal volume of stopping solution (20 nM Tris-HCl, 600 mM NaCl, 1% SDS, 10 mM EDTA) was added to stop the reaction. The final mixture was incubated at 55°C overnight with 400 µg/ml proteinase K. DNA was isolated and bisulfite converted. The regions of interest were amplified and cloned into pCR 2.1-TOPO vector (Invitrogen) for DNA sequencing.

### Hemimethylation assay

Hemimethylation analysis was performed as described previously. Undigested or Hpa II-digested DNA from RKO cells before and after treatment was subjected to bisulfite modification. Hpa II digests unmethylated DNA but does not cut a fully or hemimethylated configuration of its CCGG target sequence. Bisulfite-treated DNA was then amplified by PCR using Ms-SNuPE primers that flanked one Hpa II site at the *CDKN2A* promoter. The equations used to determine hemimethylation levels used were as described previously [25].

### Illumina Infinium DNA methylation assay (HumanMethylation27 BeadChip)

The Illumina Infinium DNA methylation assay technology has been described previously [30]. The Infinium DNA methylation assay was performed at the USC Epigenome Center according to the manufacturer's specifications (Illumina, San Diego, CA). The Illumina Infinium DNA methylation assay (HumanMethylation27_270596_v.1.2) examines DNA methylation status of 27,578 CpG sites located at promoter regions of 14,495 protein-coding genes and 110 microRNAs. Downstream processing and beta value calculations were done as previously described [53].

### Gene expression assay (Illumina HumanWG-6 v3.0 Expression BeadChip)

Expression analysis was performed using the Illumina whole-genome expression BeadChip (HumanWG-6 v3.0, 48,803 transcripts) (Illumina). The hybridized chips were stained and scanned using the Illumina HD BeadArray scanner (Illumina). Scanned image and bead-level data processing were performed using the BeadStudio 3.0.1 software (Illumina). The summarized data for each bead type were then processed using the lumi package in Bioconductor [54]. The data were log2 transformed and normalized using Robust Spline Normalization (RSN) as implemented in the lumi package.

### Statistic analysis

All statistical tests were done using R software (R version 2.12.1, 2010-12-16, R Development Core Team, 2009). ‘lumi’ package was used to normalize and process gene expression data. ‘samr’ (version 1.28) package was used for all permutation tests to access significance of gene expression changes. Differential gene expression (significance) change was established for each application by setting the cut-off on a FDR of q = 0.05 after applying 1000 permutation. The following CRAN packages were used to generate plots: ‘ggplot2’ and ‘LSD’ (version 1.0). The H2A.Z ChIP results from three biological experiments were analyzed by one way ANOVA using Prism 3(GraphPad).

### Accession numbers

All summarized probe profile data and processed expression data and DNA methylation data which are used in this study have been deposited to Gene Expression Omnibus (http://www.ncbi.nlm.nih.gov/projects/geo/) under accession Number GSE26685.

## Supporting Information

Figure S1 5-Aza-CdR treatment produces asymmetrically methylated DNA duplexes. A. ChIP results of RNA pol II enrichment at the indicated time points after 5-Aza-CdR treatment are shown. B. The schematic of working mechanism of hemimethylation assay. (U), unmethylated DNA; (H), hemimethylated DNA; (F), fully methylated DNA. Arrows indicate the Ms-SNuPE PCR primers. Open and filled circles represent unmethylated and methylated CpG sites. C. Levels of hemimethylated (H), fully methylated (F), and unmethylated (U) DNA at the Hpa II site of the *CDKN2A* promoter after 5-Aza-CdR treatment are shown. Values are expressed as relative percentages; error bars, the SD of three independent determinations. D. Agarose gel pictures showing the PCR amplicons for Hemimethylation assay. Msp I treated DNA was used as control showing the efficiency of enzyme digestion.
(TIF)

Figure S2 The CpG methylation status of the specifically amplified demethylated DNA single strands Arrows indicate TSSs and the upper vertical bars represent CpG sites. Open and filled circles represent unmethylated and methylated CpG sites respectively. Data represent the CpG methylation status of the DNA single strands shown in Figure 3.
(TIF)

Figure S3 SRCAP mediated H2A.Z deposition has minimal effects on constitutively active genes. A. RT-PCR results of the SRCAP mRNA levels in RKO cells show the knockdown efficiency of siRNA treatments. B. The methylation levels at the *MLH1*, *CDKN2A* and *MYOD1* promoters after SRCAP knockdown were measured by Ms-SNuPE in RKO cells. Error bars represent the range between biological duplicates. C. NOMe-seq results show the nucleosome occupancy levels at the *GRP78* promoters after the indicated treatments in RKO cells. D, E. The gene expression levels of *LAMB3* and *G3BP* in RKO cells were measured by RT-PCR. The data represent the means of biological triplicates. The enrichments of histone marks after SRCAP knockdown were measured by ChIP and normalized to Histone H3 levels. The data represent biological duplicates. F, G NOMe-seq results show the nucleosome occupancy at the *LAMB3* and *G3BP* promoters after SRCAP knockdown in RKO cells.
(TIF)

Figure S4 The expression levels and chromatin configurations of constitutively active genes are not disrupted by SRCAP knockdown in LD419 cells. A. The mRNA levels of the indicated genes after SRCAP knockdown in LD419 cells were measured by RT-PCR. Seventy-two hours after SRCAP siRNA treatment, the enrichments of the histone marks at *MLH1* and *CDKN2A* promoters were investigated by ChIP. Error bars represent the range between technical duplicates. B. The nucleosome occupancy at the *MLH1* promoter was detected by NOMe-seq in LD419 cells treated with the indicated siRNA for 72 hours.
(TIF)

Figure S5 DNA methylation and gene expression changes globally after 5-Aza-CdR treatment and SRCAP knockdown. A,B. Scatter plot over-layed with histogram and density distribution. Each dot represents an interrogated CpG probes beta value. Colored dots represents density or number of probes as indicated in the adjacent axis as illustrated as a both a histogram and density distribution. Contour lines are drawn to further illustrate the number of probes for a specified region. A. Represents a scatter plot between 5-Aza-CdR vs PBS treatment (control) in SRCAP siRNA treated RKO cells. B. Represents a scatter plot between SRCAP siRNA vs NC siRNA treated cells after 5-Aza-CdR treatment. C, D. Permutation results showing the number of transcripts significantly expressed as determined by the delta-cutoff. Results are presented as a Q-Q plot. C. analysis between 5-Aza-CdR vs PBS in NC siRNA treated cells. D. analysis between 5-Aza-CdR vs PBS in SRCAP siRNA treated cells. E. The gene expression log2 fold difference is plotted on the x-axis, and the q-value which accesses significance is plotted on the y-axis (−1* log10 scale). Probes that are identified as significantly different between two groups are colored in red. (5-Aza-CdR vs PBS in SRCAP siRNA treated cells) F. Venn diagram showing the number of differentially expressed genes (or transcripts) overlapping each pair of analysis.
(TIF)

Figure S6 H2A.Z is required for 5-Aza-CdR induced gene re-expression. A, B, C. The mRNA levels of the indicated genes were measured by RT-PCR to validate the genome-wide expression array results. The enrichments of histone marks at *CHFR* and *SYCP3*promoter were measured by ChIP and normalized to Histone H3 levels after the indicated treatment. The data represent biological duplicates. D, E. RKO cells were treated as indicated, and the mRNA levels of the indicated genes detected by RT-PCR as shown. Error bars represent the range between technical duplicates. F. NOMe-seq results show the nucleosome occupancy at the *CDKN2A* promoter after the indicated treatment. Green bars presents regions of 250 bp in length, which covers the −1 nucleosome plus 100 bp downstream of that nucleosome.
(TIF)
