# Supplementary figures and images for: Gene Reactivation by 5-Aza-2′-Deoxycytidine–Induced Demethylation Requires SRCAP–Mediated H2A.Z Insertion to Establish Nucleosome Depleted Regions
Source: PLoS Genet. 2012 Mar 29;8(3):e1002604. doi: 10.1371/journal.pgen.1002604 (PMC3315468; doi:10.1371/journal.pgen.1002604)

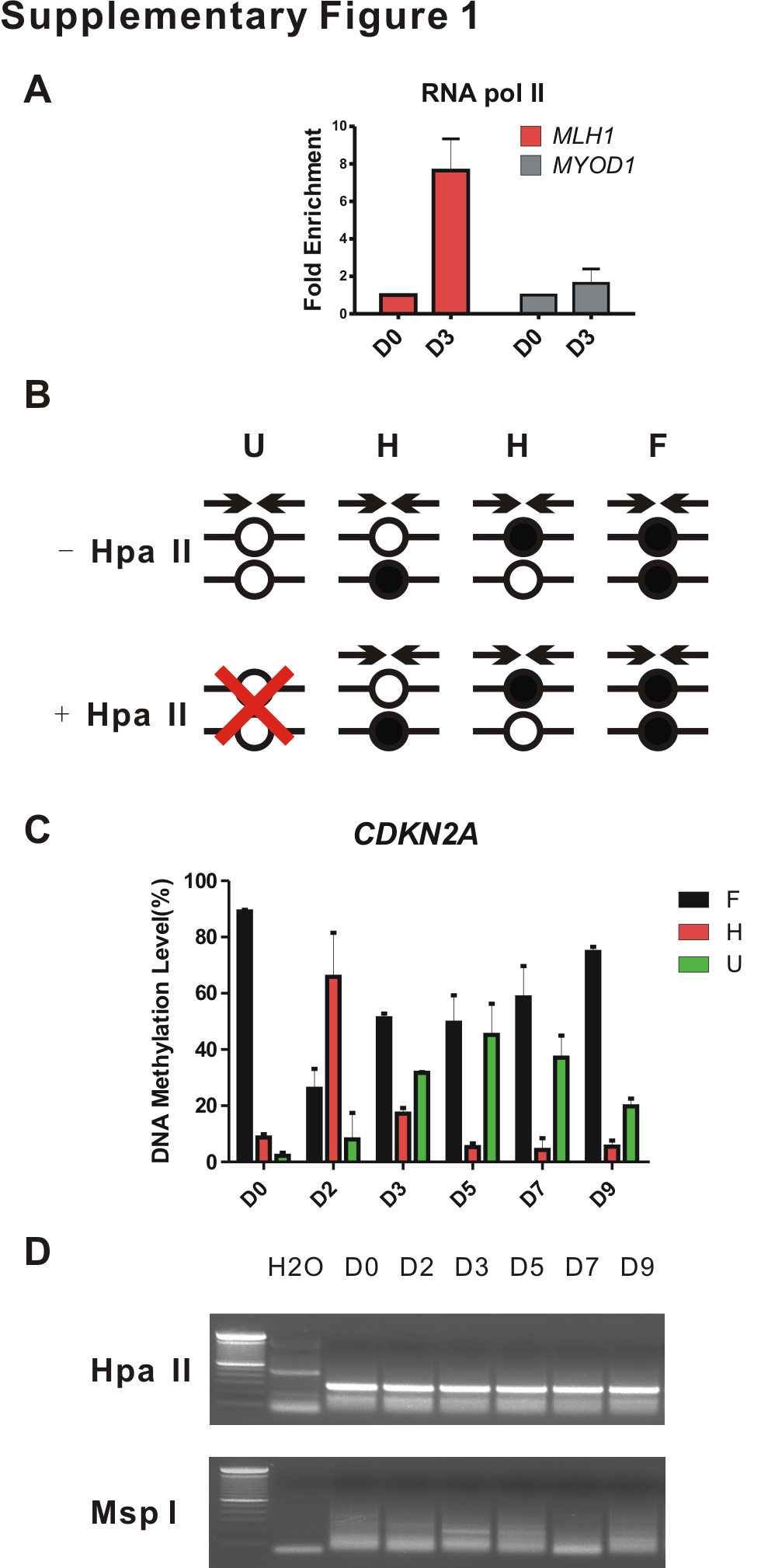

Supplement: Figure S1 — 5-Aza-CdR treatment produces asymmetrically methylated DNA duplexes. A. ChIP results of RNA pol II enrichment at the indicated time points after 5-Aza-CdR treatment are shown. B. The schematic of working mechanism of hemimethylation assay. (U), unmethylated DNA; (H), hemimethylated DNA; (F), fully methylated DNA. Arrows indicate the Ms-SNuPE PCR primers. Open and filled circles represent unmethylated and methylated CpG sites. C. Levels of hemimethylated (H), fully methylated (F), and unmethylated (U) DNA at the Hpa II site of the CDKN2A promoter after 5-Aza-CdR treatment are shown. Values are expressed as relative percentages; error bars, the SD of three independent determinations. D. Agarose gel pictures showing the PCR amplicons for Hemimethylation assay. Msp I treated DNA was used as control showing the efficiency of enzyme digestion. (TIF) [file pgen.1002604.s001.tif]

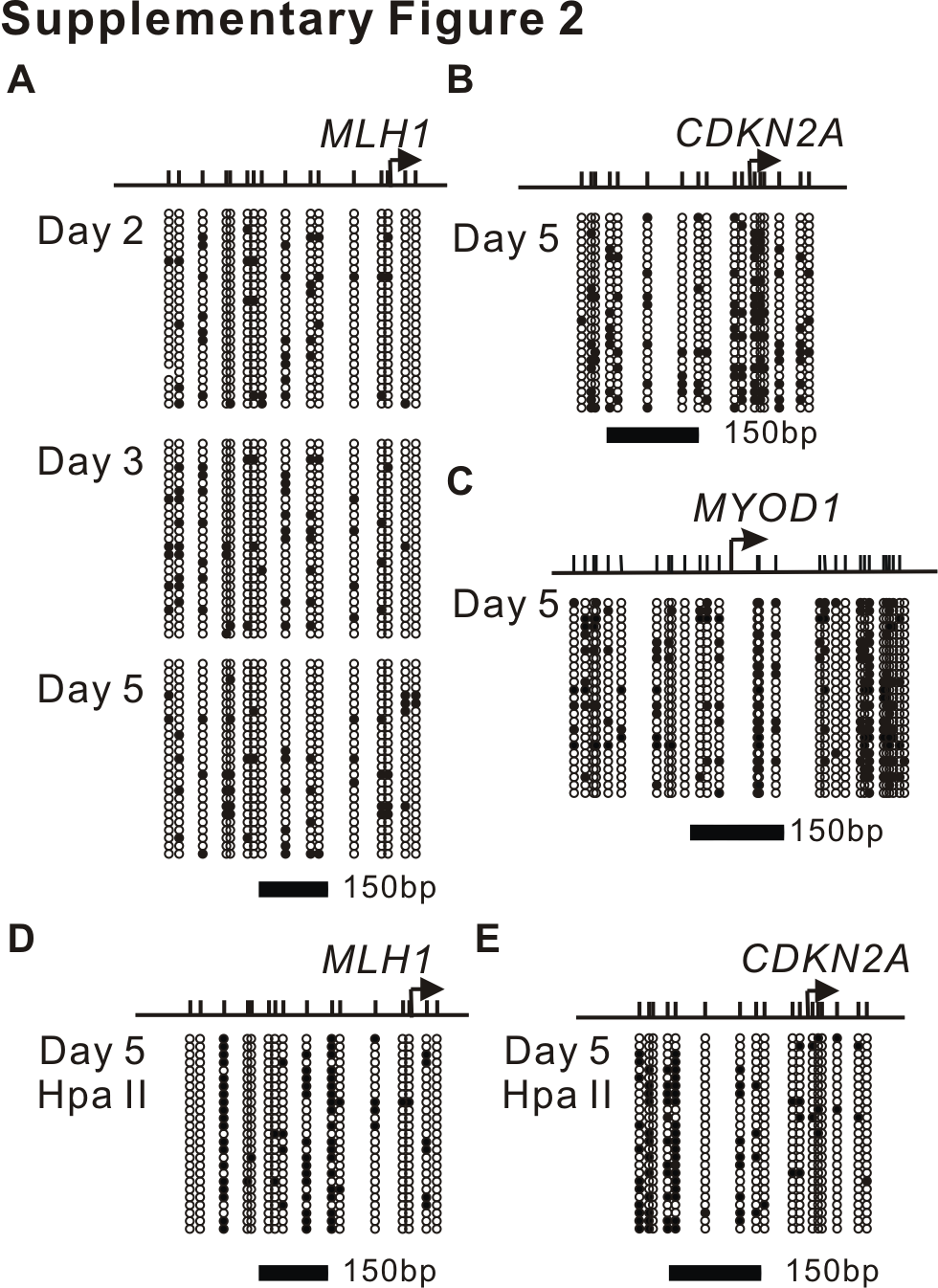

Supplement: Figure S2 — The CpG methylation status of the specifically amplified demethylated DNA single strands Arrows indicate TSSs and the upper vertical bars represent CpG sites. Open and filled circles represent unmethylated and methylated CpG sites respectively. Data represent the CpG methylation status of the DNA single strands shown in Figure 3. (TIF) [file pgen.1002604.s002.tif]

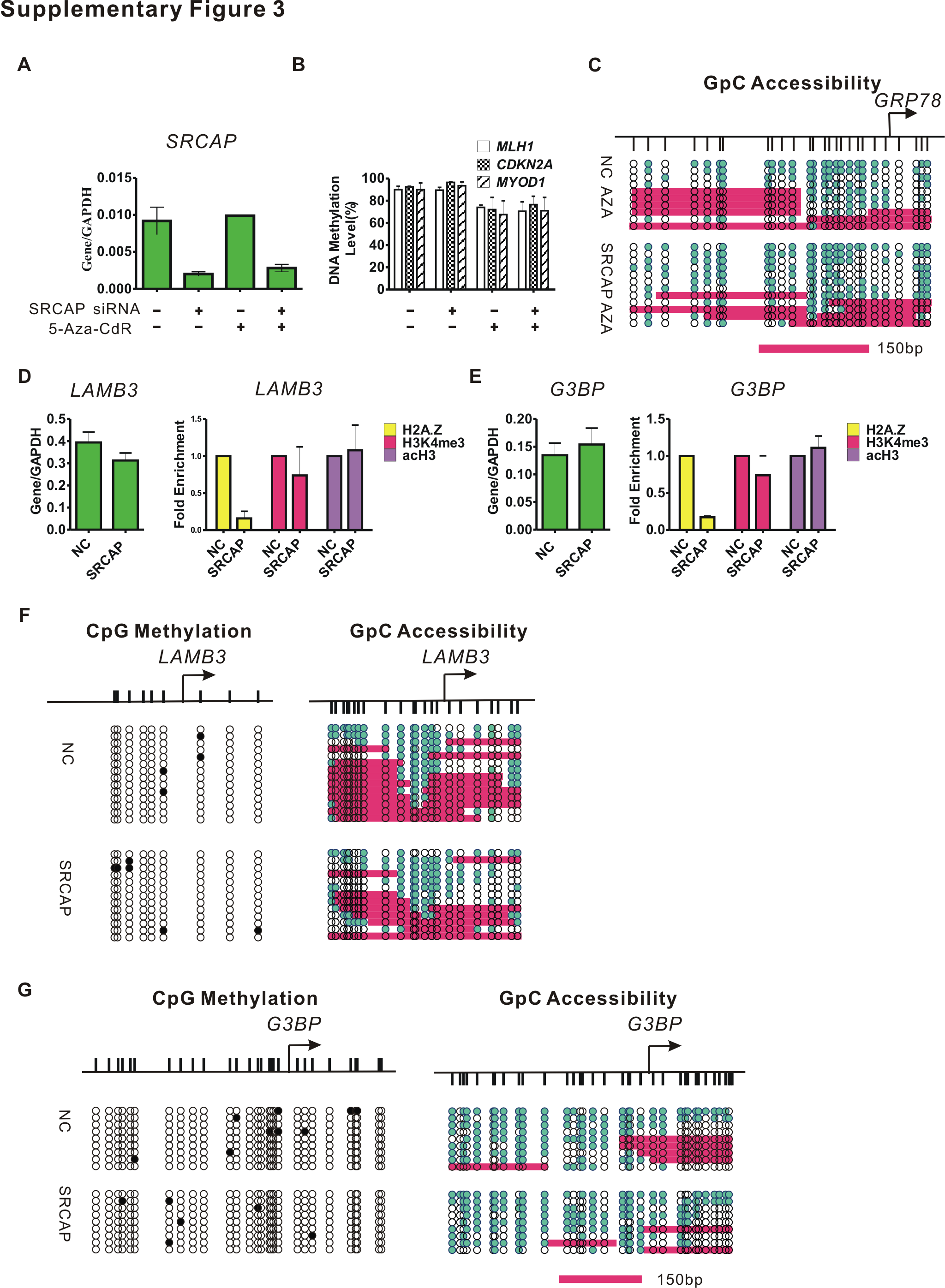

Supplement: Figure S3 — SRCAP mediated H2A.Z deposition has minimal effects on constitutively active genes. A. RT-PCR results of the SRCAP mRNA levels in RKO cells show the knockdown efficiency of siRNA treatments. B. The methylation levels at the MLH1, CDKN2A and MYOD1 promoters after SRCAP knockdown were measured by Ms-SNuPE in RKO cells. Error bars represent the range between biological duplicates. C. NOMe-seq results show the nucleosome occupancy levels at the GRP78 promoters after the indicated treatments in RKO cells. D, E. The gene expression levels of LAMB3 and G3BP in RKO cells were measured by RT-PCR. The data represent the means of biological triplicates. The enrichments of histone marks after SRCAP knockdown were measured by ChIP and normalized to Histone H3 levels. The data represent biological duplicates. F, G NOMe-seq results show the nucleosome occupancy at the LAMB3 and G3BP promoters after SRCAP knockdown in RKO cells. (TIF) [file pgen.1002604.s003.tif]

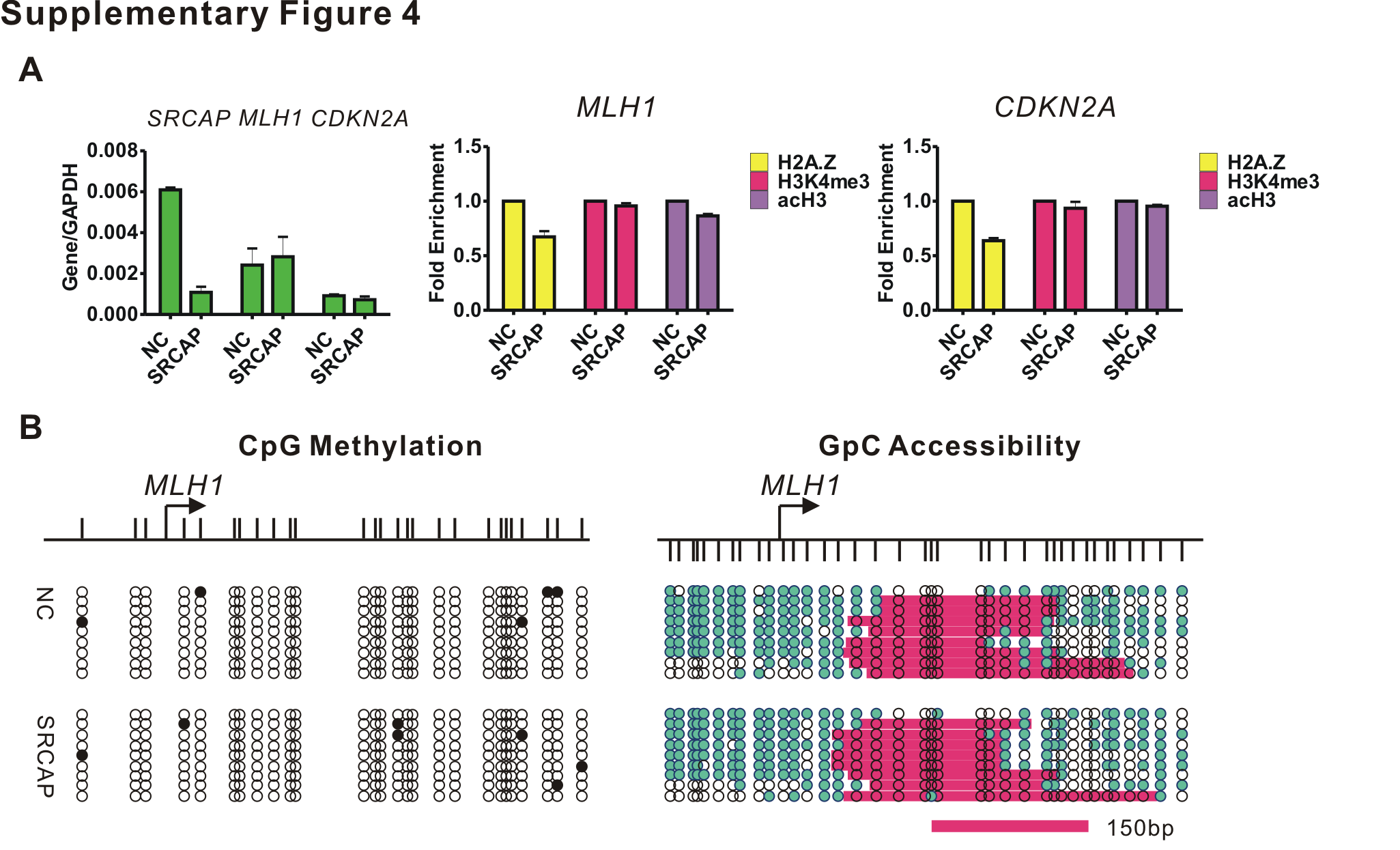

Supplement: Figure S4 — The expression levels and chromatin configurations of constitutively active genes are not disrupted by SRCAP knockdown in LD419 cells. A. The mRNA levels of the indicated genes after SRCAP knockdown in LD419 cells were measured by RT-PCR. Seventy-two hours after SRCAP siRNA treatment, the enrichments of the histone marks at MLH1 and CDKN2A promoters were investigated by ChIP. Error bars represent the range between technical duplicates. B. The nucleosome occupancy at the MLH1 promoter was detected by NOMe-seq in LD419 cells treated with the indicated siRNA for 72 hours. (TIF) [file pgen.1002604.s004.tif]

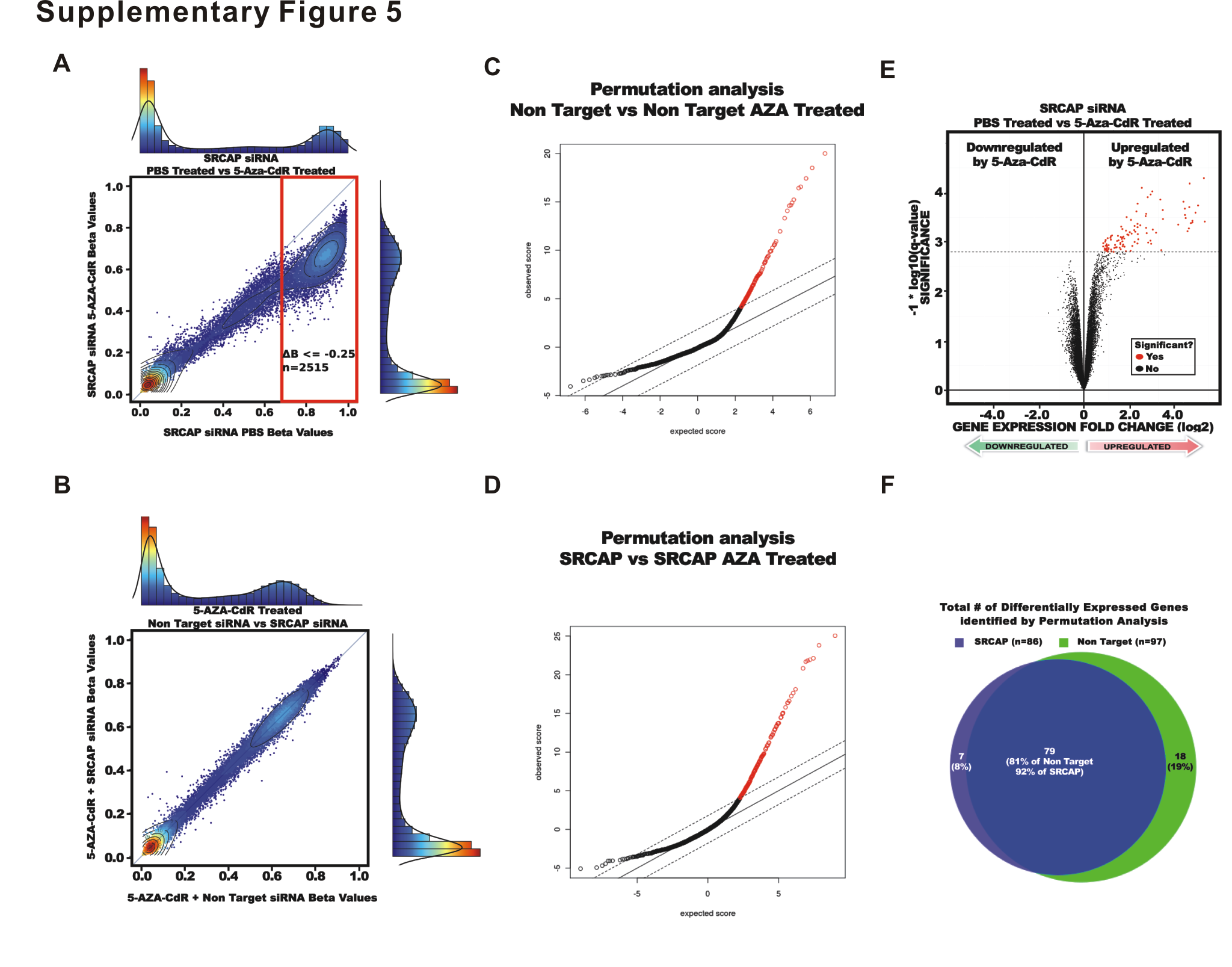

Supplement: Figure S5 — DNA methylation and gene expression changes globally after 5-Aza-CdR treatment and SRCAP knockdown. A,B. Scatter plot over-layed with histogram and density distribution. Each dot represents an interrogated CpG probes beta value. Colored dots represents density or number of probes as indicated in the adjacent axis as illustrated as a both a histogram and density distribution. Contour lines are drawn to further illustrate the number of probes for a specified region. A. Represents a scatter plot between 5-Aza-CdR vs PBS treatment (control) in SRCAP siRNA treated RKO cells. B. Represents a scatter plot between SRCAP siRNA vs NC siRNA treated cells after 5-Aza-CdR treatment. C, D. Permutation results showing the number of transcripts significantly expressed as determined by the delta-cutoff. Results are presented as a Q-Q plot. C. analysis between 5-Aza-CdR vs PBS in NC siRNA treated cells. D. analysis between 5-Aza-CdR vs PBS in SRCAP siRNA treated cells. E. The gene expression log2 fold difference is plotted on the x-axis, and the q-value which accesses significance is plotted on the y-axis (−1* log10 scale). Probes that are identified as significantly different between two groups are colored in red. (5-Aza-CdR vs PBS in SRCAP siRNA treated cells) F. Venn diagram showing the number of differentially expressed genes (or transcripts) overlapping each pair of analysis. (TIF) [file pgen.1002604.s005.tif]

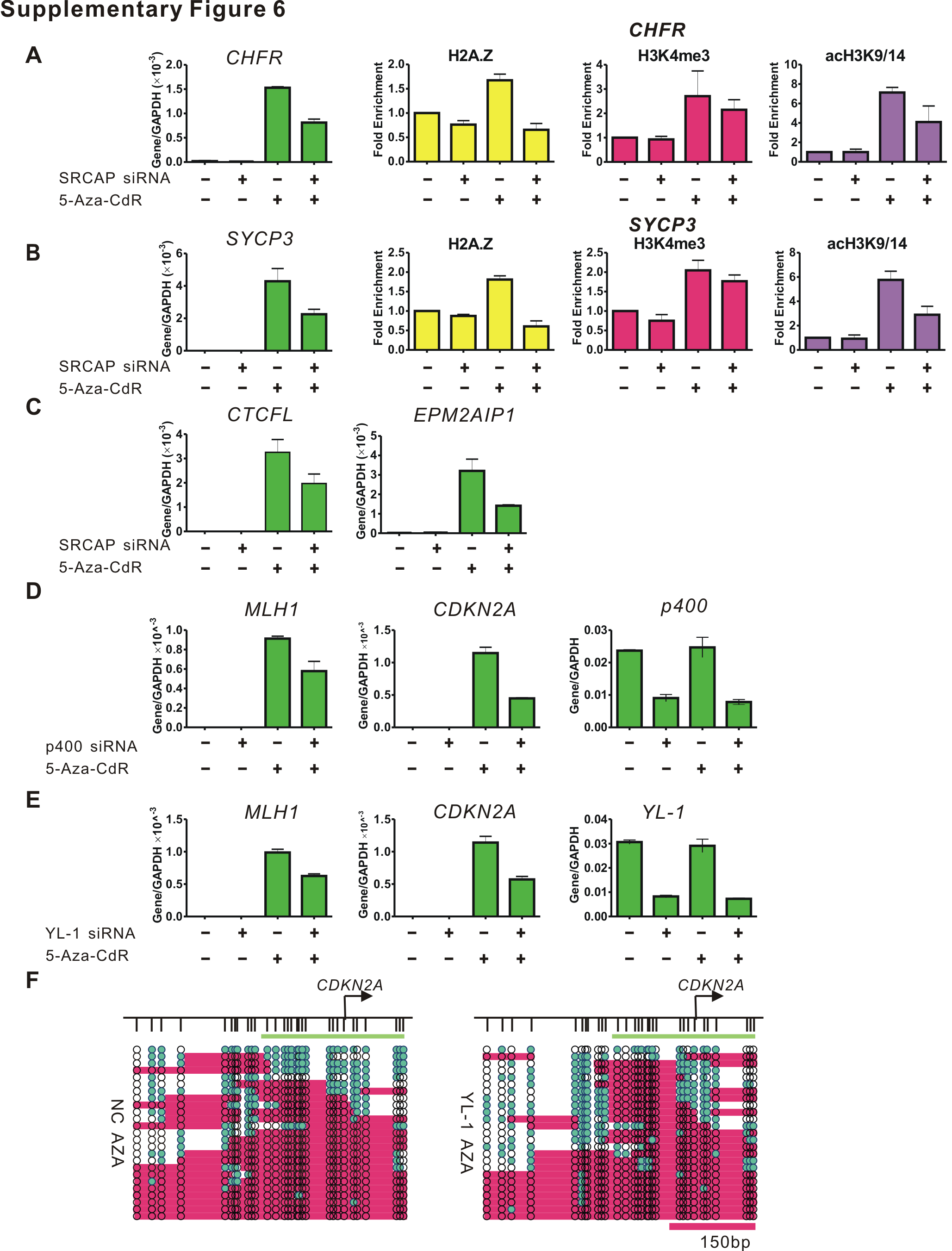

Supplement: Figure S6 — H2A.Z is required for 5-Aza-CdR induced gene re-expression. A, B, C. The mRNA levels of the indicated genes were measured by RT-PCR to validate the genome-wide expression array results. The enrichments of histone marks at CHFR and SYCP3promoter were measured by ChIP and normalized to Histone H3 levels after the indicated treatment. The data represent biological duplicates. D, E. RKO cells were treated as indicated, and the mRNA levels of the indicated genes detected by RT-PCR as shown. Error bars represent the range between technical duplicates. F. NOMe-seq results show the nucleosome occupancy at the CDKN2A promoter after the indicated treatment. Green bars presents regions of 250 bp in length, which covers the −1 nucleosome plus 100 bp downstream of that nucleosome. (TIF) [file pgen.1002604.s006.tif]
